# Deciphering the Genetic Underpinnings of Liver Cirrhosis–Heart Failure Comorbidity Through Multi-Omics: CRIM1 as a Key Endothelial Mediator

**DOI:** 10.3390/ijms27177936

**Published:** 2026-09-06

**Authors:** Ruiqi Zhao, Jiesheng Guo, Mengyao Han, Shiqi Tang, Hui Hu, Mengqing Ma, Jialing Sun, Xiaozhou Zhou

**Affiliations:** 1 The Fourth Clinical Medical College, Guangzhou University of Chinese Medicine, Shenzhen 518033, China; 20252120304@stu.gzucm.edu.cn (R.Z.);; 2 The Second Clinical Medical College, Guangzhou University of Chinese Medicine, Guangzhou 510006, China; 3 Faculty of Chinese Medicine, Macau University of Science and Technology, Taipa, Macao 999078, China

**Keywords:** liver cirrhosis, heart failure, comorbidity, GWAS, single-cell transcriptomics, endothelial cells, CRIM1, multi-omics

## Abstract

The co-occurrence of liver cirrhosis (LC) and heart failure (HF) poses considerable clinical challenges, yet the cellular and molecular determinants of this comorbidity remain poorly characterized. To address this, we developed an integrative multi-omics pipeline encompassing GWAS meta-analysis, gsMap-based spatial transcriptomic projection, GeneEnrich functional annotation, single-cell atlas construction, seismicGWAS and ECLIPSER cell-type scoring, eCAVIAR and fastenloc colocalization, hdWGCNA network inference, scTenifoldKnk in silico gene perturbation, and GCTA-COJO fine-mapping. Quality-controlled meta-analysis yielded 12,347,758 and 9,256,862 variant-level associations for LC and HF, respectively. Spatial projection confirmed preferential enrichment of disease signals within embryonic hepatic and cardiac compartments. Pathway analyses disclosed that LC-linked loci were concentrated in lipid metabolic programs, whereas HF-linked loci implicated mitochondrial bioenergetics and lysosomal degradation. At the cellular level, endothelial cells emerged as the dominant HF-associated population. Convergent evidence from five orthogonal algorithms pinpointed CRIM1 as the sole robustly supported shared gene, selectively enriched in HF endothelial cells; virtual perturbation further identified LCP1 and PTPRC as downstream regulatory nodes. Fine-mapping of the chromosome 2 locus harboring rs12476437 revealed multiple statistically independent signals in the vicinity of CRIM1. Collectively, these findings computationally prioritize the endothelial–CRIM1 axis as a previously unappreciated candidate mechanistic bridge between LC and HF requiring experimental validation.

## 1. Introduction

Liver cirrhosis (LC) represents the shared end-stage pathological destination of virtually all forms of chronic liver disease, defined histologically by diffuse parenchymal fibrosis, regenerative nodule formation, and profound disruption of hepatic architecture, culminating in portal hypertension and progressive liver failure [1]. On a global scale, LC imposes a substantial and growing public health burden, with both prevalence and mortality continuing to rise [2]. Heart failure (HF), in turn, is a complex clinical syndrome rooted in impaired cardiac pump function that renders peripheral tissues chronically underperfused; affecting over 64 million individuals worldwide, it carries a five-year mortality exceeding 50%, rendering it one of the most formidable threats to human health [3,4].

Critically, LC and HF frequently converge in the same patient, bound together by a bidirectional and self-reinforcing pathological relationship. On one hand, the portal hypertension and systemic hemodynamic derangements wrought by LC—including hyperdynamic circulation and avid sodium and water retention—directly compromise cardiac structure and function, a phenomenon recognized clinically as cirrhotic cardiomyopathy [5]. On the other hand, the chronic venous congestion imposed by HF, most pronounced in the setting of right ventricular failure, produces sustained passive hepatic congestion, which may give rise to congestive hepatopathy or accelerate fibrotic progression in pre-existing liver disease [6]. This state of comorbidity substantially amplifies the risk of adverse outcomes and renders clinical management especially demanding. Nevertheless, a systematic dissection of the shared pathological mechanisms driving LC–HF comorbidity at the level of genetics and molecular biology has, until now, remained conspicuously absent from the literature.

From a pathophysiological standpoint, both LC and HF are characterized by the convergent dysfunction of multiple organ systems. Chronic inflammation, oxidative stress, metabolic dysregulation, and vascular endothelial injury represent common pathological threads woven through both conditions [5,7]. Positioned at the interface between the circulation and peripheral tissues, vascular endothelial cells are indispensable guardians of vascular homeostasis, modulators of inflammatory responses, and mediators of inter-organ signaling [8]. Endothelial dysfunction contributes to portal hypertension and hepatic sinusoidal remodeling in LC [9], while in HF it is intimately linked to coronary microvascular impairment and maladaptive ventricular remodeling [10]. Endothelial cells may therefore represent a critical cellular nexus bridging the comorbid mechanisms of LC and HF.

Genome-wide association studies (GWAS) have proven to be powerful instruments for mapping the genetic underpinnings of complex diseases. Independent GWAS have already identified multiple risk loci for LC and HF in isolation [11,12], yet a systematic investigation of their shared genetic architecture has not been undertaken. A fundamental challenge is that the vast majority of GWAS-implicated variants reside in non-coding genomic regions, making direct inference of causal genes and effector cell types elusive—a limitation that has catalyzed the development of numerous methods for integrating functional genomic data with GWAS signals [13]. Colocalization of expression quantitative trait loci (eQTL) and splicing quantitative trait loci (sQTL) with GWAS signals offers a principled route to nominating candidate causal genes by linking genetic associations to transcriptional regulation [14].

The rapid maturation of single-cell RNA sequencing (scRNA-seq) has opened unprecedented opportunities to decode disease-associated genetic signals at the resolution of individual cell types [15]. By marrying GWAS summary statistics with single-cell transcriptomic data, emerging algorithms such as seismicGWAS [16] and ECLIPSER [17,18] enable systematic identification of cell populations in which genetic risk is preferentially concentrated, thereby illuminating the principal effector cells of disease. Concurrently, hdWGCNA [19]—a single-cell-adapted implementation of weighted gene co-expression network analysis—facilitates the discovery of functionally coherent gene modules within specific cell populations, deepening our understanding of disease-associated transcriptional programs. Beyond these tools, gsMap leverages spatial transcriptomic data to anchor GWAS signals within anatomically defined tissue compartments, providing a developmental biology perspective on disease-relevant tissues [20].

CRIM1 (Cysteine-Rich Transmembrane BMP Regulator 1) encodes a single-pass type I transmembrane protein haboring cysteine-rich von Willebrand factor A domains, and is recognized as a key modulator of bone morphogenetic protein (BMP) signaling, with established roles in vascular development and the maintenance of endothelial cell integrity [21]. Prior work has demonstrated that CRIM1 is highly expressed in vascular endothelial cells, where it governs endothelial survival, migration, and angiogenic responses [22]. Yet its potential contributions to the pathogenesis of LC and HF, and the genetic mechanisms that regulate it in these contexts, have not been systematically explored.

Against this backdrop, the present study set out to construct a multi-dimensional integrative framework spanning GWAS, spatial transcriptomics, and single-cell RNA sequencing to systematically dissect the shared genetic and molecular basis of LC–HF comorbidity. At the tissue level, gsMap spatial mapping was employed to anchor disease-enriched anatomical compartments. At the cellular level, seismicGWAS and ECLIPSER were applied to single-cell transcriptomic data integrated with GWAS signals to prioritize candidate pathogenic cell types. At the gene level, eCAVIAR and fastenloc colocalization analyses together with hdWGCNA co-expression network analysis were deployed in cross-validated fashion to nominate core causal genes, complemented by scTenifoldKnk virtual knockout to interrogate their regulatory networks. At the variant level, GCTA-COJO conditional analysis was used to resolve independent association signals within implicated loci. Through this strategy, we sought to identify the core effector cell type and master regulatory gene of LC–HF comorbidity, provide computational genetic evidence illuminating the molecular basis of cardiac–hepatic crosstalk, and lay the groundwork for the rational design of comorbidity-directed therapeutic interventions.

## 2. Results

### 2.1. Genome-Wide Meta-Analysis of LC and HF

By pooling GWAS summary statistics across two independent cohorts for each condition, we successfully amplified the statistical power to detect disease-associated genetic variants. Following rigorous quality control, the meta-analysis yielded 12,347,758 genome-wide loci for LC and 9,256,862 for HF.

### 2.2. Quality Control of GWAS Data

**Figure 1:**
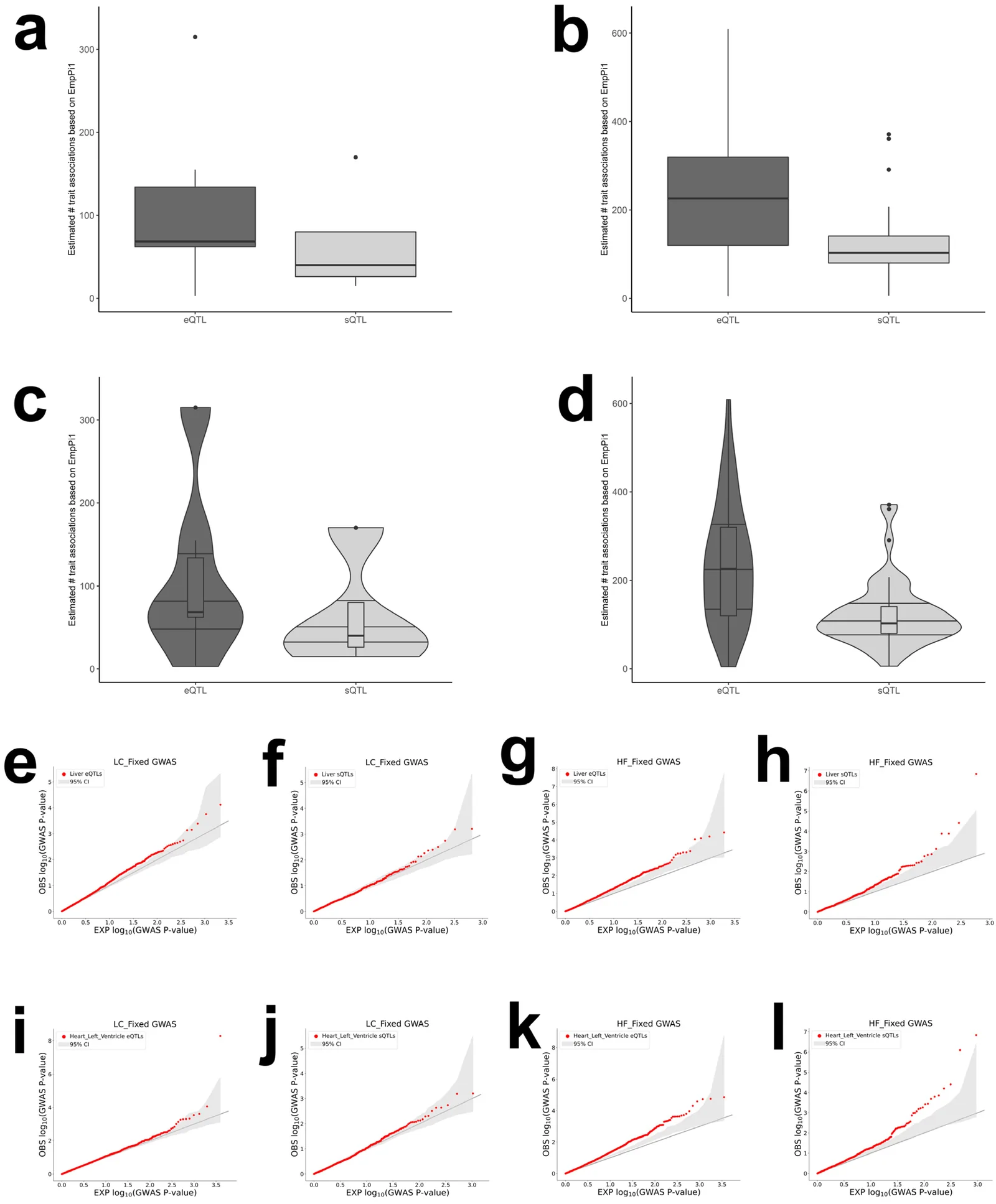
QTL enrichment analysis and genome-wide association landscapes for LC and HF. (**a**,**b**) Box plots depicting the distribution of QTLEnrich statistics for (**a**) LC and (**b**) HF. Dark gray denotes expression quantitative trait loci (eQTLs) and light gray denotes splicing quantitative trait loci (sQTLs). (**c**,**d**) Violin plots showing the distribution of QTLEnrich statistics for (**c**) LC and (**d**) HF. Dark gray denotes eQTLs and light gray denotes sQTLs. (**e**) Quantile–quantile (QQ) plot of SNP-level *p* values for eQTLs in liver tissue from the LC GWAS dataset. The *x*-axis represents the expected distribution of *p* values under the null hypothesis, and the *y*-axis represents the observed distribution. The gray shaded area indicates the expected null distribution with its 95% confidence interval. (**f**) QQ plot of SNP-level *p* values for sQTLs in liver tissue from the LC GWAS dataset; axes and shading are as described in (**e**). (**g**) QQ plot of SNP-level *p* values for eQTLs in liver tissue from the HF GWAS dataset; axes and shading are as described in (**e**). (**h**) QQ plot of SNP-level *p* values for sQTLs in liver tissue from the HF GWAS dataset; axes and shading are as described in (**e**). (**i**) QQ plot of SNP-level *p* values for eQTLs in heart tissue from the LC GWAS dataset; axes and shading are as described in (**e**). (**j**) QQ plot of SNP-level *p* values for sQTLs in heart tissue from the LC GWAS dataset; axes and shading are as described in (**e**). (**k**) QQ plot of SNP-level *p* values for eQTLs in heart tissue from the HF GWAS dataset; axes and shading are as described in (**e**). (**l**) QQ plot of SNP-level *p* values for sQTLs in heart tissue from the HF GWAS dataset; axes and shading are as described in (**e**).

The reliability of the e/sQTL data incorporated into downstream analyses was validated through complementary graphical diagnostics—boxplots, violin plots, and quantile–quantile (QQ) plots—all of which confirmed robust data quality (Figure 1a–l).

**Figure 2:**
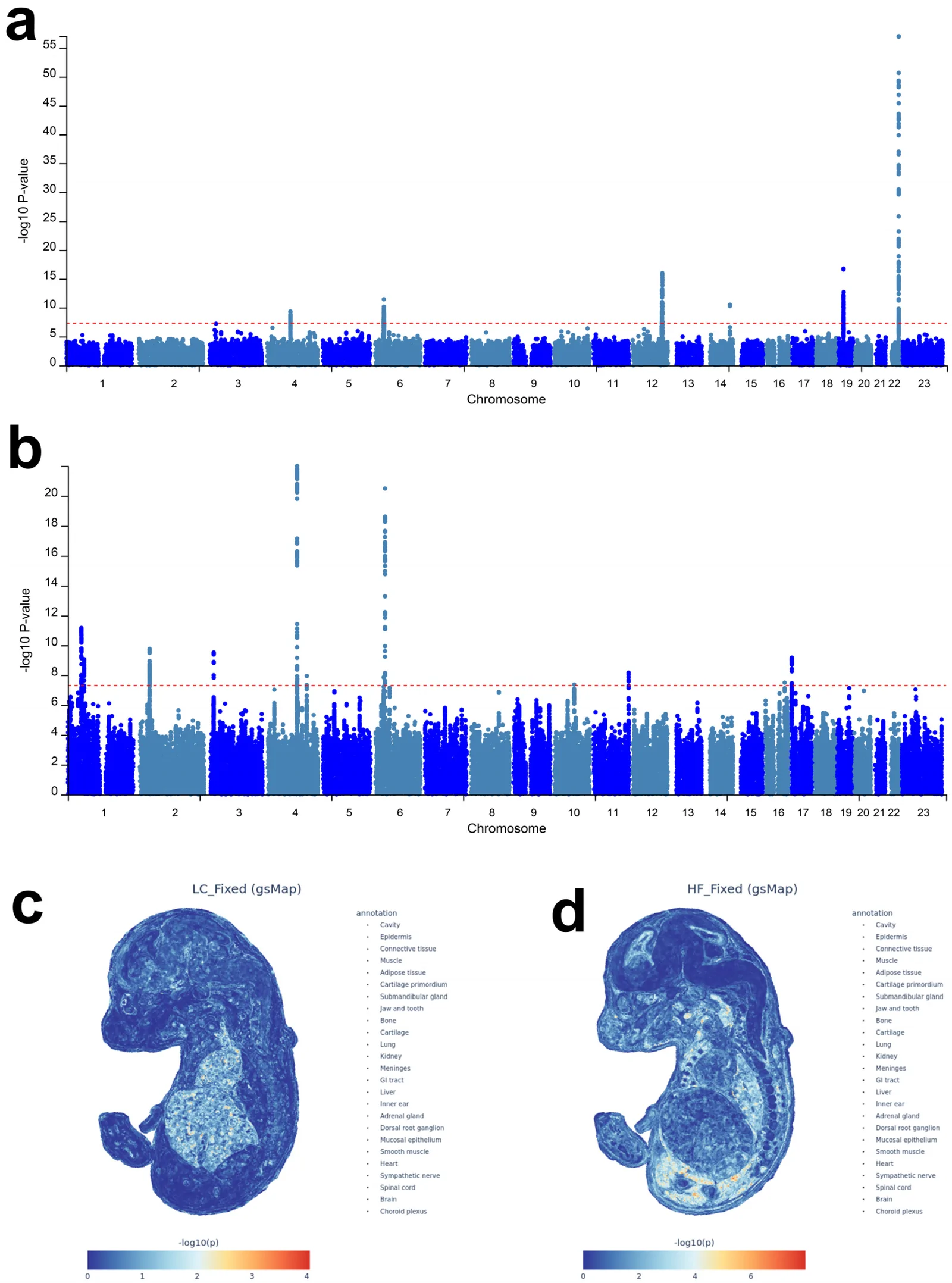
Tissue-level spatial mapping of genetic signals for LC and HF. (**a**) Manhattan plot of the LC GWAS data. The red dashed line indicates the genome-wide significance threshold (*p* = 5 × 10^−8^). (**b**) Manhattan plot of the HF GWAS data. The red dashed line indicates the genome-wide significance threshold (*p* = 5 × 10^−8^). (**c**) Spatial distribution map of LC-associated genetic signals projected onto E16.5 mouse embryo data using the gsMap algorithm. Redder points indicate smaller *p* values. (**d**) Spatial distribution map of HF-associated genetic signals projected onto E16.5 mouse embryo data using the gsMap algorithm. Redder points indicate smaller *p* values.

Manhattan plots for both LC (Figure 2a) and HF (Figure 2b) revealed prominent association peaks spanning multiple chromosomes, consistent with the polygenic architecture of each condition.

### 2.3. Spatial Transcriptomic Mapping Delineates the Tissue Basis of LC and HF

gsMap successfully projected the GWAS genetic signals of LC and HF onto the spatial transcriptomic atlas of E16.5 mouse embryonic tissue, with statistically significant enrichment observed in both hepatic and cardiac compartments (LC: Liver, *P*_Cauchy = 0.040; Heart, *P*_Cauchy = 0.026; HF: Liver, *P*_Cauchy = 0.034; Heart, *P*_Cauchy = 0.041) (Figure 2c,d). These findings delineate the spatial pathological substrate of each condition and provide an anatomically grounded perspective on their shared disease mechanisms.

### 2.4. Gene Set Enrichment Analysis Reveals Divergent Biological Programs

**Figure 3:**
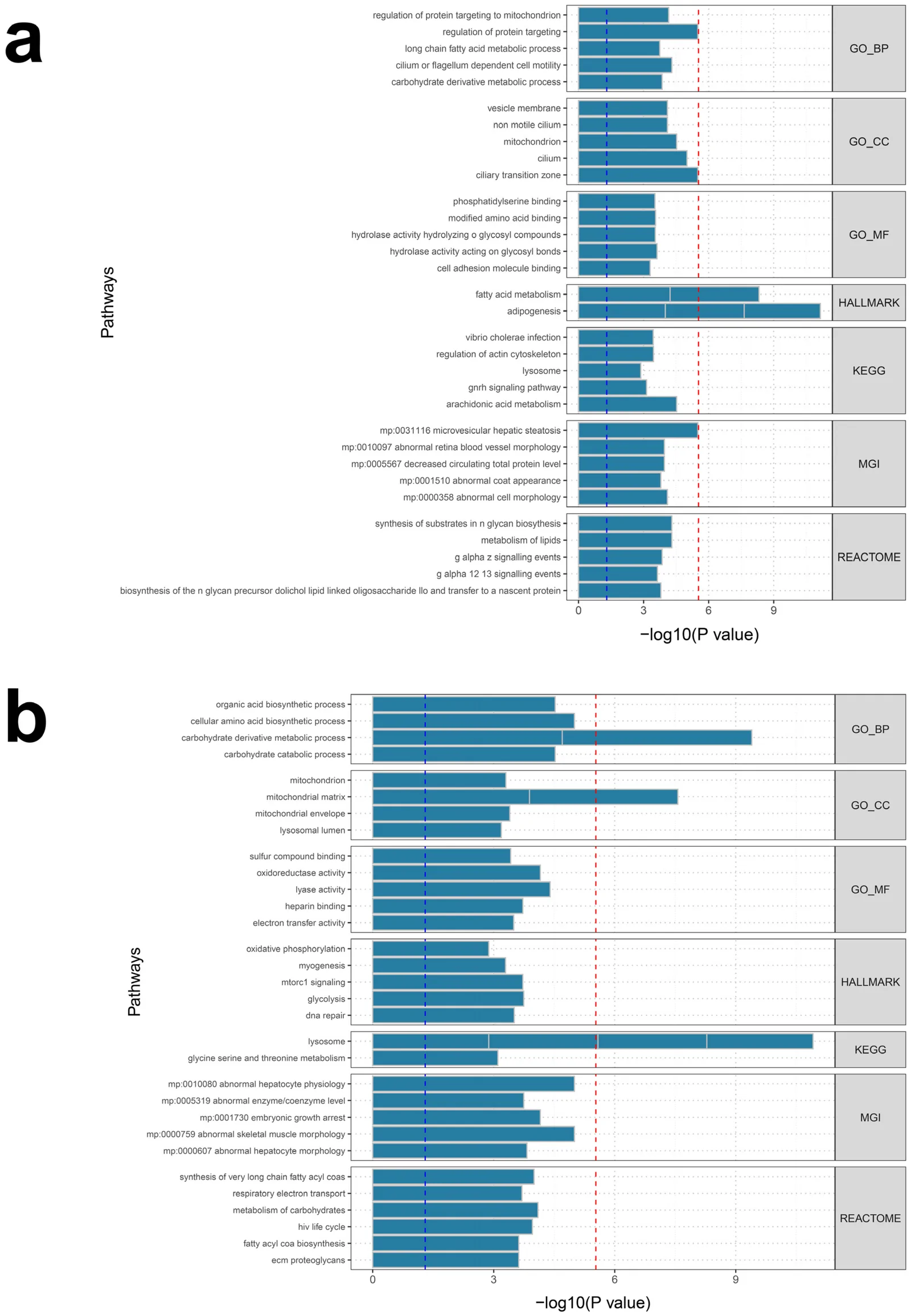
Pathway enrichment analysis of disease-associated eGenes and sGenes. (**a**) Pathway enrichment results of eGenes and sGenes significantly associated with LC across multiple gene sets. (**b**) Pathway enrichment results of eGenes and sGenes significantly associated with HF across multiple gene sets. The *x*-axis represents −log_10_(*p* value). The blue dashed line indicates *p* = 0.05 and the red dashed line indicates *p* = 1 × 10^−5^. The *y*-axis represents the corresponding pathways.

To systematically characterize the biological functions encoded within the LC- and HF-associated genetic signals, GeneEnrich was applied to evaluate pathway-level enrichment among significantly associated genes (full results in Supplementary Table S1). Genes associated with LC were significantly enriched for fatty acid metabolism (HALLMARK; empirical_*p*val < 1 × 10^−5^) and adipogenesis (HALLMARK; empirical_*p*val < 1 × 10^−5^) (Figure 3a). In contrast, HF-associated genes converged on carbohydrate derivative metabolic process (GO BP; empirical_*p*val < 1 × 10^−5^), mitochondrial matrix (GO CC; empirical_*p*val < 1 × 10^−5^), and lysosome (KEGG; empirical_*p*val < 1 × 10^−5^) (Figure 3b). This divergence in pathway enrichment suggests that LC and HF, despite their clinical comorbidity, engage distinct molecular programs within their respective target organs.

### 2.5. Cellular Composition of LC and HF

**Figure 4:**
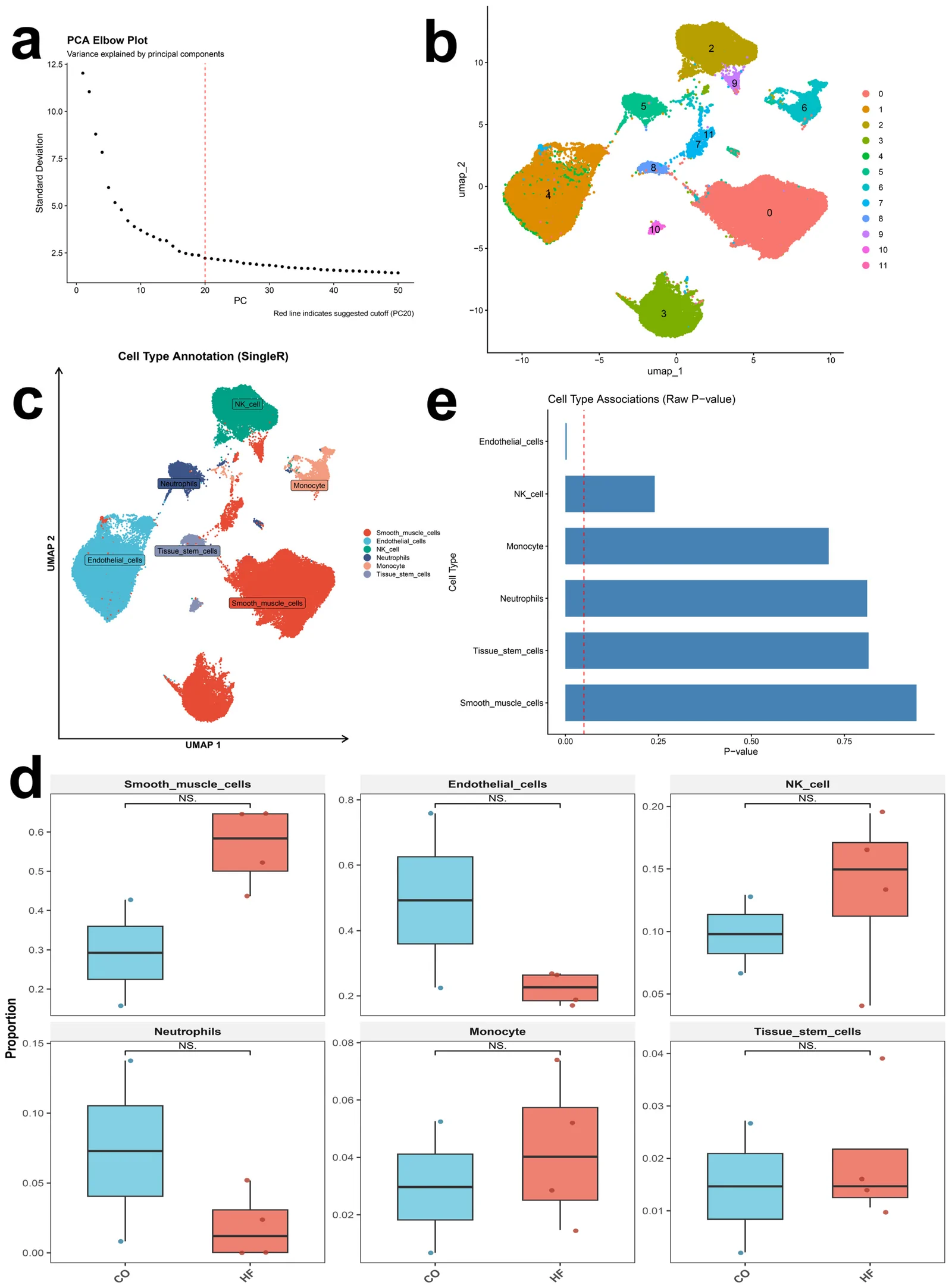
Single-cell transcriptomic characterization. (**a**) Principal component analysis (PCA) elbowplots of single-cell transcriptomic data. (**b**) Uniform Manifold Approximation and Projection (UMAP) visualization of single-cell transcriptomic data. (**c**) UMAP plots following cell type annotation for single-cell transcriptomic data. (**d**) Box plots depicting cell type proportion differences in single-cell transcriptomic data. Blue represents the control group and orange represents the disease group. Statistical significance was assessed using the Wilcoxon rank-sum test. NS., *p* > 0.05. (**e**) Bar plot showing cell type prioritization results from seismicGWAS analysis of single-cell transcriptomic data. The red dashed line indicates *p* = 0.05.

Building upon data that had undergone rigorous QC, normalization, and Harmony-based batch correction, unsupervised clustering and cell type identification were performed on the HF single-cell dataset—noting that the LC single-cell data had been filtered to retain hepatocytes exclusively. The dimensionality of the data was first assessed using an elbow plot (Figure 4a).

A cell neighborhood graph was subsequently constructed on the Harmony-corrected low-dimensional embedding, and multi-resolution clustering was applied; clustering tree analysis identified the optimal resolution yielding robust and reproducible cell populations (Figure 4b). Automated annotation via SingleR against curated reference datasets resolved six major cell types in the HF atlas: smooth muscle cells, endothelial cells, NK cells, neutrophils, monocytes, and tissue stem cells (Figure 4c).

Wilcoxon rank-sum tests comparing per-sample cell type proportions between disease and control groups revealed no statistically significant differences in the abundance of any cell type (Figure 4d).

ECLIPSER-based cell-type enrichment analysis did not identify any cell type reaching statistical significance for either LC or HF (Table 1). In contrast, seismicGWAS identified endothelial cells as significantly associated with HF (*p* < 0.05) (Figure 4e).

### 2.6. Co-Expression Network Analysis in Endothelial Cells

**Figure 5:**
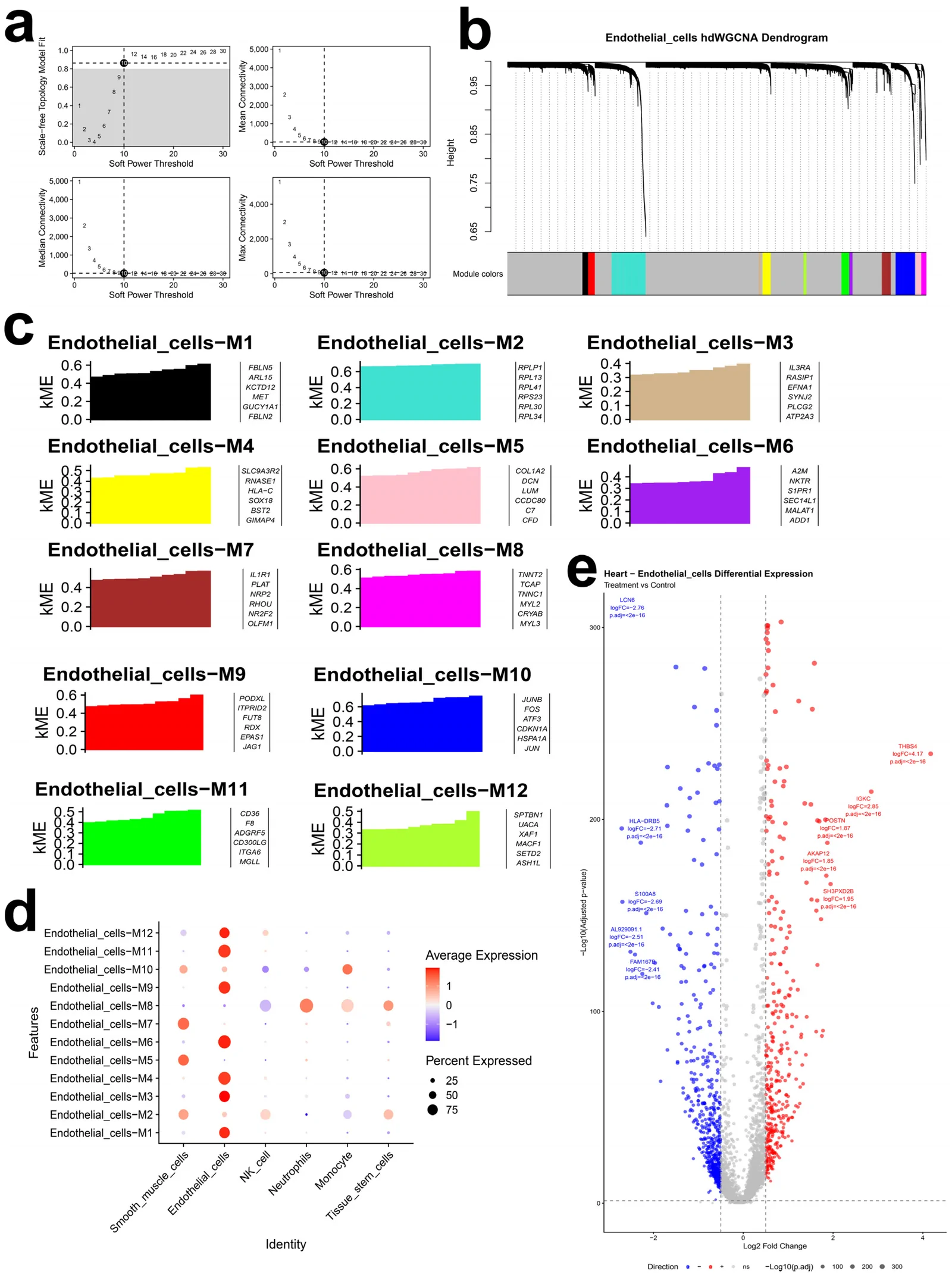
Co-expression network analysis and differential gene analysis. (**a**) Network topology plots displaying scale-free fitces across a range of soft-thresholding powers from hdWGCNA analysis of single-cell transcriptomic data. (**b**) Co-expression network construction based on endothelial cells, showing the optimal soft threshold and dendrogram with module color assignments. (**c**) Module Eigengene of endothelial cells. (**d**) Dot plots illustrating the expression profiles of distinct gene modules derived from endothelial cells across different cell types in single-cell transcriptomic data. (**e**) Differential gene volcano map of endothelial cells.

Converging multi-dimensional evidence from the analyses described above pointed to endothelial cells as a candidate pathogenic cell type in LC–HF comorbidity. To characterize the transcriptional regulatory landscape of endothelial cells in this disease context, hdWGCNA was applied. To overcome inherent data sparsity, metacells were constructed (k = 25), and a soft-thresholding power of 10 (Figure 5a) was selected to build a signed topological overlap matrix (TOM) approximating a scale-free network topology.

Dynamic tree cutting of the resulting hierarchical clustering tree delineated multiple co-expression modules (Figure 5b), each characterized by a set of high-ranking hub genes (Figure 5c). Dot plots depicting the expression of endothelial cell modules across all annotated cell types revealed that multiple modules displayed elevated expression in the HF dataset (Figure 5d). Finally, differential expression analysis between control and disease endothelial cells was visualized as a volcano plot, with significantly upregulated genes in the disease group highlighted in red and downregulated genes in blue (Figure 5e).

eCAVIAR successfully nominated multiple putative causal genes and regulatory variants shared between LC and HF. Complementary fastenloc analysis, applied to GWAS loci against eQTL/sQTL signals across 49 GTEx tissues (v8), further identified genes with high regional colocalization probabilities. The complete colocalization results are provided in Supplementary Table S2.

### 2.7. Cell-Type Expression Annotation and Exon-Level Splicing Analysis of the Prioritized Gene

**Figure 6:**
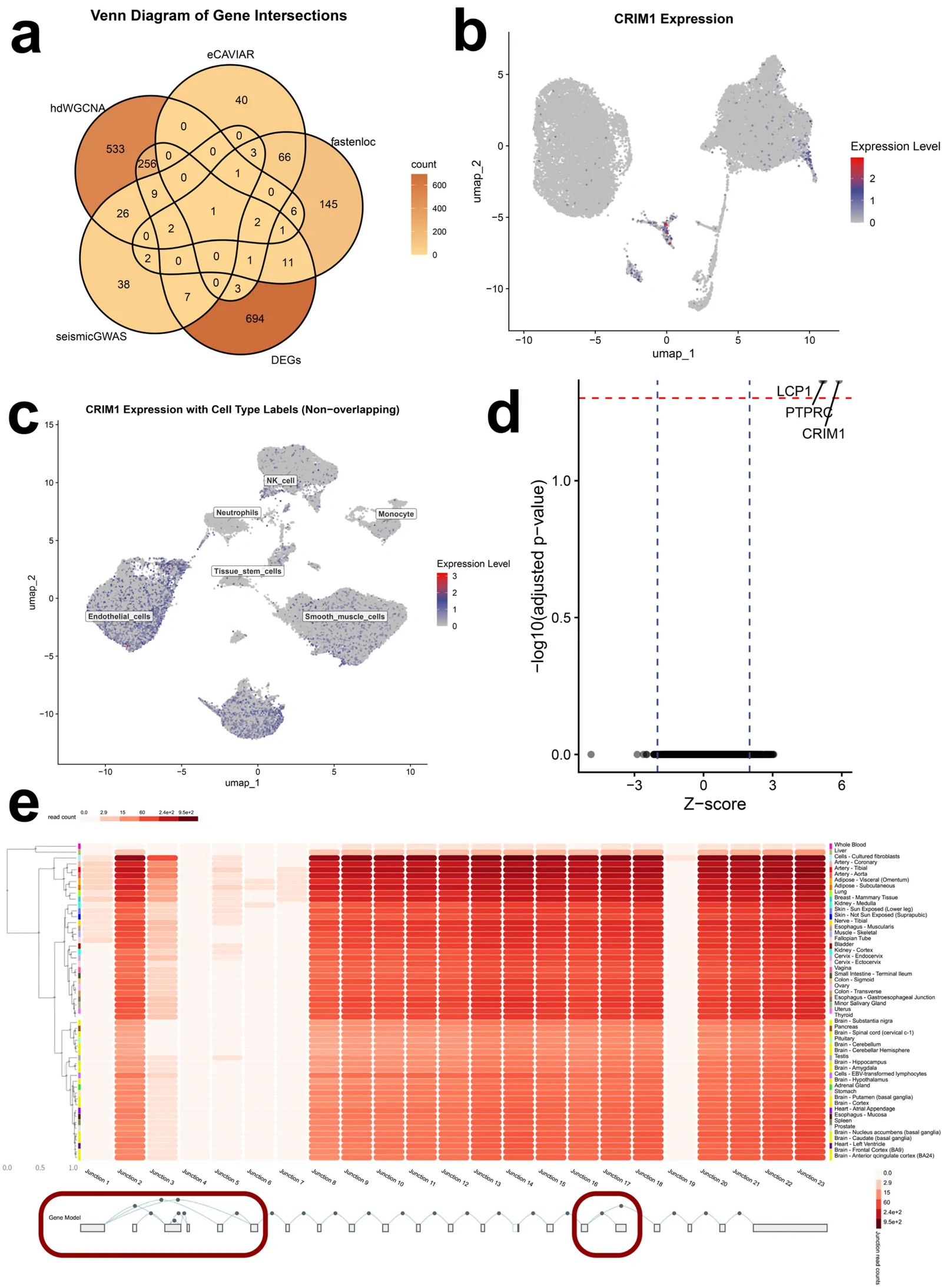
Identification and functional characterization of CRIM1 as a convergent key gene. (**a**) Venn diagram depicting the intersection of key genes identified by five analytical approaches. (**b**) UMAP plot of CRIM1 expression in LC single-cell transcriptomic data; redder color indicates higher expression levels. (**c**) UMAP plot of CRIM1 expression in HF single-cell transcriptomic data; redder color indicates higher expression levels. (**d**) Volcano map after virtual knockout of CRIM1 gene. (**e**) Model of the CRIM1 gene. Connecting lines represent splicing events between all exons. The red box highlights the putative alternative splicing site. The heatmap above displays tissue-level expression of the gene, with deeper red indicating higher expression. The figure panel was adapted from the GTEx Portal (https://gtexportal.org/; accessed 16 May 2026).

To resolve the cellular origin of the key pathogenic gene, evidence from all five analytical algorithms was integrated by taking their intersection (Figure 6a; Supplementary Table S3). This cross-algorithm convergence nominated a single high-confidence candidate: CRIM1. In the LC single-cell transcriptomic data, UMAP visualization demonstrated uniformly low CRIM1 expression in hepatocytes (Figure 6b). In the HF dataset, CRIM1 was prominently expressed in endothelial cells, with markedly lower expression across all other cell types (Figure 6c).

Virtual knockout of CRIM1 via scTenifoldKnk revealed that, beyond CRIM1 itself, LCP1 and PTPRC were the most substantially perturbed genes, whereas no other genes showed appreciable disruption—implicating LCP1 and PTPRC as key regulatory partners of CRIM1 (Figure 6d). It is essential to emphasize that these predicted regulatory relationships are derived entirely from computational network inference and require experimental validation. scTenifoldKnk virtual knockout infers gene-gene dependencies from transcriptional network topology changes but cannot distinguish direct regulatory interactions from indirect cascades or correlated expression. Experimental validation through CRIM1 knockdown or knockout in endothelial cells, followed by transcriptomic profiling or targeted qPCR/Western blotting of LCP1 and PTPRC, would be necessary to confirm these predicted dependencies and elucidate the underlying molecular mechanisms.

Exon-level analysis indicated that differential CRIM1 expression may be attributable to alternative splicing events in the Junction 1–6 and 16–17 regions (Figure 6e).

### 2.8. Genomic Risk Locus Architecture

Integrating the colocalization evidence described above [23], one high-risk variant was identified: rs12476437, located on chromosome 2 in proximity to the CRIM1 gene. Subsequent GCTA-COJO conditional analysis, conditioning on rs12476437, tested 44 variants within the surrounding locus for independent association (Supplementary Table S4). Five variants—rs114309145, rs10490673, rs58456579, rs58919832, and rs6713312—remained significant after conditioning (p_conditional < 0.05), indicating the presence of multiple independent association signals within this locus and suggesting that several functionally distinct variants independently contribute to disease risk at this genomic region.

## 3. Discussion

Through the construction of a systematic multi-omics integration framework, this study dissected the shared genetic and molecular architecture of LC–HF comorbidity across tissue, cellular, and molecular strata. Two overarching conclusions emerge: endothelial cells represent a candidate effector cell type through which HF genetic risk is transduced, and may serve as a pivotal cellular bridge in the LC–HF comorbid relationship; and CRIM1, corroborated by five independent analytical algorithms, stands as a high-confidence shared candidate gene whose genomic locus harbors multiple independent functional variants.

gsMap successfully mapped the GWAS genetic signals of both LC and HF onto the spatial transcriptomic atlas of E16.5 mouse embryos, with statistically significant enrichment concurrently observed in hepatic and cardiac tissue compartments. This concordant spatial projection furnishes genetic evidence, framed within a developmental biology context, for the shared tissue basis of LC–HF comorbidity, suggesting that the genetic risk architecture of both diseases may have left a common transcriptional imprint on hepatic and cardiac tissue as early as the embryonic stage.

From a pathophysiological perspective, the bidirectional organ crosstalk between LC and HF is profound. The hyperdynamic circulatory state, systemic arterial vasodilation, and neurohumoral dysregulation—including hyperactivation of the renin–angiotensin–aldosterone system—that accompany LC can directly precipitate structural and functional cardiac changes [1,24]. Epidemiological data indicate that the prevalence of HF among patients with decompensated LC substantially exceeds that in the general population, and that the presence of HF independently worsens the prognosis of LC patients [4]. Conversely, the elevated right-sided filling pressures and systemic venous hypertension imposed by HF are transmitted via the hepatic veins, producing passive hepatic congestion, centrilobular fibrosis, and, in extreme cases, congestive hepatic fibrosis [6]. This reciprocal organ interaction creates a vicious cycle in which each condition accelerates the progression of the other, and our genomic findings provide molecular-level evidence for this intimate inter-organ linkage.

GeneEnrich pathway enrichment analysis further illuminated the divergent biological mechanisms of LC and HF. LC-associated genetic signals were prominently enriched for fatty acid metabolism and adipogenesis, in close alignment with the central pathological role of hepatocellular lipid metabolic imbalance, hepatic steatosis, and fibrotic progression in LC [25]. HF-associated signals, by contrast, converged on carbohydrate derivative metabolism, mitochondrial matrix, and lysosome gene sets, implicating impaired cardiomyocyte energy metabolism, mitochondrial dysfunction, and dysregulated autophagy as core biological processes in HF genetics [26]. This divergence in pathway enrichment underscores the notion that, despite their clinical co-occurrence, LC and HF likely engage distinct molecular mechanisms within their respective target organs, while endothelial cells may serve as a common effector interface that integrates pathological signals from these disparate upstream pathways.

seismicGWAS identified endothelial cells as the cell type most significantly enriched for HF genetic risk (*p* < 0.05). By incorporating a specificity score that simultaneously reflects expression level and cross-cell consistency, seismicGWAS circumvents the arbitrary threshold setting that hampers many alternative approaches, conferring statistical robustness. Although ECLIPSER did not identify any cell type reaching significance in the present datasets—a finding plausibly attributable to the relatively modest number of genome-wide significant GWAS loci, which may have limited the power of its locus-based Bayesian enrichment estimator—the evidence from seismicGWAS aligns compellingly with the well-established centrality of endothelial cells in cardiovascular pathology, and the deployment of both complementary methods strengthens the overall inference.

Endothelial dysfunction is widely regarded as a pivotal pathological mechanism in both LC and HF [8]. Within the hepatic microenvironment of LC, the phenotypic transformation—capillarization—and functional deterioration of liver sinusoidal endothelial cells (LSECs) are recognized as critical initiating events in portal hypertension and hepatic fibrogenesis [9]. Under physiological conditions, LSECs sustain a low-resistance sinusoidal milieu largely through constitutive nitric oxide (NO) production; in LC, however, attenuated NO biosynthesis combined with augmented endothelin-1 secretion conspires to elevate intrahepatic vascular resistance [10]. In HF, dysfunction of coronary microvascular endothelial cells propagates ventricular remodeling through oxidative stress, inflammatory activation, and extracellular matrix reorganization, with prognostic consequences for affected patients [27].

From a systemic vantage point, endothelial cells lining the vascular beds of every organ constitute the biological interface through which the circulation communicates with peripheral tissues. Patients with LC exhibit pervasive systemic endothelial dysfunction—manifesting as impaired endothelium-dependent vasodilation, heightened vascular permeability, and a prothrombotic state—that extends beyond the portal circulation to encompass the coronary vasculature, thereby forging a direct endothelial link between LC and HF [28]. Our multi-omics analysis provides independent genomic evidence for the central position of endothelial cells in LC–HF comorbidity.

Cross-referencing the outputs of five independent analytical pipelines—eCAVIAR colocalization, fastenloc colocalization, hdWGCNA co-expression network analysis, differential expression analysis, and seismicGWAS—converged on CRIM1 emerges as a computationally prioritized shared candidate gene. CRIM1 (Cysteine-Rich Transmembrane BMP Regulator 1) encodes a type I single-pass transmembrane protein bearing cysteine-rich von Willebrand factor A domains, and functions as an important modulator of bone morphogenetic protein (BMP) signaling [29]. Through its extracellular domain, CRIM1 engages multiple BMP ligands and tethers them to the cell surface, thereby fine-tuning the spatial and temporal distribution of BMP signaling intensity [30].

At the level of cellular expression, our single-cell transcriptomic data show that CRIM1 is prominently expressed in endothelial cells in HF, with markedly lower expression across other cell populations; in the LC hepatocyte dataset, CRIM1 expression was uniformly low. This cell-type-restricted expression pattern is consistent with CRIM1’s established role in vascular endothelial biology [22]. Prior studies have documented high CRIM1 expression in glomerular capillary endothelial cells and retinal vascular endothelium, where it governs endothelial survival signaling and angiogenic programs [21]. Crim1-deficient mouse models develop severe renal microvascular defects characterized by loss of capillary integrity and increased endothelial apoptosis [21], underscoring the indispensable contribution of CRIM1 to the structural maintenance of the microvasculature.

BMP signaling exerts broad regulatory influence over cardiac and vascular homeostasis. Ligands including BMP-2, BMP-4, and BMP-9 engage endothelial receptor complexes to govern endothelial proliferation, differentiation, migration, and survival [31]. Within the heart, BMP signaling participates in the regulation of myocardial hypertrophy, and perturbation of this pathway—whether through aberrant activation or suppression—is implicated in the pathogenesis of HF [32]. In the liver, the BMP/SMAD axis plays a dual role in regulating systemic iron homeostasis and hepatic fibrotic responses [33]. Positioned as a regulatory node within BMP signaling, CRIM1 may therefore simultaneously influence the pathological processes unfolding in both the heart and the liver by modulating the fidelity of BMP signal transduction in endothelial cells—providing a biologically coherent rationale for its emergence as a shared comorbidity candidate gene.

The results of scTenifoldKnk virtual knockout shed additional light on the gene regulatory network in which CRIM1 operates. Following in silico ablation of CRIM1, LCP1 (Lymphocyte Cytosol Protein 1) and PTPRC (protein tyrosine phosphatase receptor type C, also known as CD45) were the most substantially perturbed genes, while the remainder of the network remained largely undisturbed. LCP1 (L-plastin) is an actin-bundling protein that orchestrates cytoskeletal dynamics and migratory behavior in immune cells [34]. PTPRC (CD45) is a hallmark surface glycoprotein of hematopoietic cells that serves as an essential protein tyrosine phosphatase in lymphocyte signal transduction and activation [35]. The marked perturbation of these two genes upon CRIM1 ablation raises the intriguing possibility that CRIM1 participates in the regulation of endothelial–immune cell crosstalk at the vascular interface—a hypothesis that merits rigorous experimental validation.

Exon-level analysis further revealed that differential CRIM1 expression may be attributable, at least in part, to alternative splicing events within the Junction 1–6 and 16–17 regions. Alternative splicing is a pervasive mechanism through which the mammalian genome diversifies its proteome, with distinct isoforms often exhibiting markedly divergent functional properties [36]. The tissue-specific splicing landscape of CRIM1 and its functional implications across hepatic and cardiac contexts warrant dedicated investigation in future studies.

Colocalization analysis pinpointed rs12476437 on chromosome 2, in proximity to CRIM1, as the lead risk variant at this locus. This localization is wholly consistent with the cellular and gene-level evidence accumulated throughout this study, further consolidating CRIM1’s candidacy as a shared disease gene. As the lead variant within this region, rs12476437 may exert its pathogenic effects through cis-regulatory mechanisms that alter CRIM1 transcription or splicing, thereby perturbing the fine-tuned BMP signaling balance in endothelial cells and contributing to the shared pathogenesis of LC and HF.

GCTA-COJO conditional analysis, conditioning on rs12476437, revealed that five additional variants within the locus—rs114309145, rs10490673, rs58456579, rs58919832, and rs6713312—retained genome-wide significance after conditioning (*p*_conditional < 0.05), establishing the presence of multiple independent association signals at this genomic region. This pattern of allelic heterogeneity indicates that the locus is governed by several functionally distinct variants that independently modulate disease risk, possibly through differing molecular mechanisms such as eQTL effects, sQTL effects, or disruption of cis-regulatory elements [13]. Such genetic complexity poses challenges for variant-level functional dissection and suggests that the contribution of this locus to disease risk may vary across individuals of differing genetic backgrounds.

The present findings carry several practical implications for the management of LC–HF comorbidity. The identification of endothelial cells as the core effector population lends support to positioning endothelial protection as a therapeutic priority in this comorbid setting. Several pharmacological approaches targeting endothelial function have already been explored separately in LC and HF—including the endothelial-protective properties of non-selective beta-blockers such as carvedilol and the endothelial-restoring effects of renin–angiotensin–aldosterone system inhibitors—and their cumulative benefit on endothelial integrity in the comorbid context deserves evaluation in dedicated clinical trials. Furthermore, the nomination of CRIM1 as a key candidate gene raises the possibility that the BMP signaling axis may represent a tractable warrant experimental investigation as potential therapeutic targets in LC–HF comorbidity; as pharmacological modulators of BMP signaling advance through the pipeline, there is a compelling case for exploring agents capable of simultaneously restoring endothelial function in both cardiac and hepatic vascular beds.

Several limitations of this study merit consideration. First, the GWAS datasets analyzed were predominantly derived from European-ancestry cohorts, and the generalizability of our genetic findings to populations of non-European ancestry remains uncertain. Population-specific differences in linkage disequilibrium structure, allele frequencies, and effect sizes may limit the transferability of CRIM1 locus associations and eQTL colocalization signals to African, Asian, or admixed populations. Additionally, LC and HF are etiologically heterogeneous conditions: LC may arise from viral hepatitis, alcohol, metabolic dysfunction, or autoimmune causes, while HF encompasses ischemic, non-ischemic, valvular, and infiltrative subtypes. The GWAS cohorts we analyzed aggregate across these etiological subtypes, potentially obscuring subtype-specific genetic architectures. Future studies incorporating ancestry-diverse cohorts and etiology-stratified analyses would clarify the generalizability and refine the biological interpretation of the endothelial–CRIM1 axis. Second, the single-cell transcriptomic data were relatively limited in sample size—the LC dataset comprising only one case and one control, and the HF dataset four cases and two controls—and the LC analysis was further restricted to hepatocytes, precluding a comprehensive characterization of the full cellular diversity of LC. Public data availability for LC and HF single-cell atlases remains limited compared to other diseases, underscoring the need for coordinated efforts to generate larger, multi-center single-cell reference datasets for these conditions is essential before these computational predictions can be considered robust. Public data availability for LC and HF single-cell atlases remains limited compared to other diseases, underscoring the need for coordinated efforts to generate. These constraints may have reduced statistical power for certain analyses, and replication in larger, more comprehensive single-cell cohorts will be important. And critically undermining our endothelial cell hypothesis in the LC context, the LC single-cell dataset was restricted to hepatocytes only, completely excluding non-parenchymal cell populations including liver sinusoidal endothelial cells, hepatic stellate cells, Kupffer cells, cholangiocytes, and infiltrating immune cells. This represents a limitation because our central hypothesis posits endothelial cells as the key cellular bridge between LC and HF, yet we were unable to directly assess CRIM1 expression or disease-associated transcriptional changes in liver endothelial cells. Consequently, all LC-related cellular inferences are confined to hepatocytes, and the endothelial component of our proposed LC-HF axis is supported only by the HF single-cell data. Third, this study is fundamentally computational in nature; while cross-validation across multiple orthogonal algorithms enhances the reliability of the conclusions, the precise molecular mechanisms through which CRIM1 in endothelial cells mediates LC–HF comorbidity remain to be established through in vitro functional assays, organoid models, and in vivo animal experiments. Finally, the comorbid relationship between LC and HF likely involves additional biological dimensions—including the gut microbiome, metabolome, and epigenome—that fall outside the scope of the present analysis and represent important directions for future multi-omics integration efforts.

## 4. Materials and Methods

### 4.1. Sources of Genome-Wide Summary Statistics

This study focused on publicly available GWAS summary-level data for LC and HF, incorporating two independent cohorts for each condition. GWAS data for LC were obtained from the FinnGen database (ID: CHIRHEP_NAS; 1703 cases and 494,803 controls) and the IEU Open GWAS database (ID: ebi-a-GCST90018826; 122 cases and 347,284 controls). GWAS data for HF were similarly drawn from the FinnGen database (ID: I9_HEARTFAIL; 37,653 cases and 462,695 controls) and the IEU Open GWAS database (ID: ebi-a-GCST009541; 47,309 cases and 930,014 controls).

### 4.2. Single-Cell Transcriptomic Data

Single-cell RNA sequencing data for LC were retrieved from the publicly accessible GEO repository (accession: GSE312804), comprising one LC sample and one healthy control. HF single-cell data were obtained from GEO (accession: GSE247468), encompassing four HF samples and two controls.

### 4.3. Quality Control

Prior to downstream analysis, a rigorous quality control (QC) pipeline was applied to all GWAS datasets to safeguard analytical accuracy and reliability. To ensure adequate representation of common variation and to minimize spurious associations, the minor allele frequency (MAF) was computed for each SNP; variants falling below the prespecified MAF threshold of 0.01 were excluded to reduce bias and enhance data integrity. Genome build harmonization was also performed to ensure consistent coordinate systems across datasets before proceeding to subsequent analyses.

For single-cell transcriptomic data, a systematic QC workflow was implemented using the Seurat package (version 5.0.0). Following data loading and initialization, the proportions of mitochondrial transcripts (identified by the “^MT-” prefix) and hemoglobin-encoding genes (including HBA1, HBA2, and HBB) were computed as proxies of cell viability. High-quality cells were retained by applying stringent thresholds: a minimum of 1000 unique molecular identifiers (UMIs) per cell, between 200 and 5000 detected genes per cell, mitochondrial gene content not exceeding 15%, and hemoglobin gene content not exceeding 3%. Dimensionality reduction was carried out using principal component analysis (PCA) followed by UMAP embedding. To mitigate batch effects arising from inter-sample variability, Harmony was applied to integrate data across samples prior to downstream analyses.

### 4.4. Genome-Wide Meta-Analysis

To augment statistical power for the detection of genetic variants with modest effect sizes, fixed-effects meta-analysis was performed separately for LC and HF by pooling GWAS summary statistics across the two independent cohorts for each condition.

### 4.5. Tissue-Level Enrichment via Spatial Transcriptomic Mapping

To delineate the tissue-specific anatomical basis of LC and HF, we integrated GWAS summary statistics with single-cell spatial transcriptomic (sc-ST) data using gsMap (v1.73.7) (genetically informed spatial mapping of cells for complex traits). This algorithm leverages GWAS-derived trait-associated genes and maps their expression profiles onto spatially resolved cells, thereby quantifying the association between specific anatomical compartments and complex traits at single-cell resolution. Leveraging the spatial transcriptomic atlas of E16.5 mouse embryonic tissue spanning 25 organs, we generated tissue enrichment specificity maps and spatially resolved gene expression landscapes for both LC and HF, ultimately constructing phenotypic spatial pathogenesis maps at single-cell resolution.

### 4.6. Biological Pathway Enrichment Using GeneEnrich

Gene set enrichment analysis was conducted using the GeneEnrich tool to systematically characterize the biological processes implicated by LC- and HF-associated genetic variants. Enrichment of candidate gene sets was assessed against curated functional gene sets through hypergeometric and permutation-based testing, with empirical *p* values derived from permutation procedures to mitigate tissue-specificity bias. Functional annotations were drawn from Gene Ontology, Reactome, the Kyoto Encyclopedia of Genes and Genomes (KEGG), the Molecular Signatures Database (MSigDB), and the Mouse Genome Informatics (MGI) database. A nominal significance threshold of *p* < 0.05 was applied.

### 4.7. Single-Cell Atlas Annotation

Processed single-cell data were subjected to multi-dimensional visualization using the Harmony-corrected low-dimensional embedding. Cell neighborhood graphs were constructed on the basis of the Harmony-reduced representation, and community detection was performed using a multi-resolution clustering strategy; optimal resolution was determined by inspection of the clustering tree to yield stable, reproducible cell populations. Cluster-defining marker genes were identified using the FindAllMarkers function (minimum expression fraction of 0.25 and log-fold-change threshold of 0.25), and a heatmap of top marker genes was generated to facilitate cluster characterization. Automated cell type annotation was accomplished using the SingleR algorithm against curated reference datasets. Differences in cell type proportions between disease and control groups were assessed using the Wilcoxon rank-sum test applied to per-sample proportions.

### 4.8. Cell-Type Prioritization Using seismicGWAS

The seismicGWAS framework [16] computes a novel specificity score that simultaneously captures the expression intensity and cross-cell consistency of each gene within a given cell type. Compared with alternative approaches—such as scDRS, FUMA, and S-MAGMA—that integrate GWAS with transcriptomic data, seismicGWAS offers improved robustness by sidestepping the need for arbitrary association thresholds. Specifically, GWAS summary statistics augmented with MAGMA Z-scores, together with annotated single-cell transcriptomic data, were supplied as input to the seismicGWAS R package. For each gene, the specificity score reflects two complementary properties: consistently elevated expression in the target cell type relative to all others, and broad expression across cells within that type. Trait–cell-type associations were then quantified by integrating per-gene specificity scores with MAGMA Z-scores. Cell types achieving *p* ≤ 0.05 were considered significantly associated with LC or HF.

### 4.9. Cell-Type Prioritization Using ECLIPSER

ECLIPSER [17,18] employs a Bayesian Fisher’s exact test framework, benchmarked against a background set of GWAS loci, to estimate cell-type-specific enrichment fold changes and associated *p* values for each trait–tissue–cell-type combination. The cell-type specificity threshold is defined at the 95th percentile of background locus scores. The Bayesian formulation enables estimation of 95% confidence intervals for enrichment fold changes, making it suitable even for traits with sparse loci or limited signal above the enrichment threshold; annotations are derived from a multi-tiered functional genomics platform. In practice, candidate loci were first defined from GWAS results, then expanded via linkage disequilibrium (LD) proxies (r^2^ > 0.8) to comprehensively capture potential functional variants. Cell types reaching *p* ≤ 0.05 were designated as statistically significant enriched populations.

### 4.10. hdWGCNA Co-Expression Network Analysis in Prioritized Cell Types

Weighted gene co-expression network analysis was performed on the prioritized cell type using hdWGCNA [19]. The annotated Seurat object was loaded and the target cell type specified for analysis. The WGCNA environment was initialized using the WGCNA function, and genes expressed in at least 5% of cells were retained as candidate features. Metacells were constructed via the MetacellsByGroups function (k = 25), grouping cells by cell type and sample of origin to mitigate single-cell data sparsity while preserving biological variation; metacell expression matrices were subsequently normalized and scaled. Following PCA-based dimensionality reduction, Harmony was applied to correct for sample-level batch effects. The expression matrix for the target cell type was extracted using the SetDatExpr function, and the soft-thresholding power was selected through TestSoftPowers to approximate a scale-free network topology, upon which a signed topological overlap matrix (TOM) was constructed. Co-expression modules were identified using the dynamic tree-cutting algorithm, and module eigengene values were computed. Module–cell-type association analyses were subsequently conducted.

### 4.11. Colocalization Analysis: eCAVIAR

To identify high-confidence causal genes and regulatory mechanisms (eQTL/sQTL) at loci associated with both LC and HF, we employed eCAVIAR [37], a Bayesian colocalization method that evaluates whether co-occurring GWAS and e/sQTL signals tag the same causal variant or haplotype, explicitly accounting for local LD structure and allelic heterogeneity. eCAVIAR incorporates fine-mapping functionality capable of handling large-scale GWAS summary data. A maximum of two independent causal variants per locus was assumed. Input comprised Z-scores (effect estimate divided by its standard error) from both GWAS and GTEx e/sQTL datasets. The LD window surrounding each lead variant was defined as the chromosomal region encompassing all variants with r^2^ > 0.1 (computed using 1000 Genomes Project Phase 3 as the reference panel), extended by an additional 50,000 bp on each flank. A colocalization posterior probability (CLPP) exceeding 0.01 was considered evidence of significant colocalization.

### 4.12. Colocalization Analysis: Fastenloc

Complementing eCAVIAR, we applied the Bayesian colocalization method fastenloc [38] to fine-map causal regulatory signals at LC- and HF-associated loci. fastenloc uses its embedded DAP-G algorithm to independently fine-map GWAS and e/sQTL loci, estimating the posterior inclusion probability for each variant and subsequently computing the probability that the two signals share a common causal variant, without imposing an upper bound on the number of independent causal variants per locus. Z-scores derived from GWAS and GTEx e/sQTL summary data served as input. LD windows were defined identically to those used in eCAVIAR. Colocalization results are summarized as regional colocalization probabilities (RCP); following the authors’ recommendations, an RCP > 0.1 was taken as evidence of significant colocalization.

### 4.13. Virtual Knockout Using scTenifoldKnk

scTenifoldKnk [39] is a computationally efficient virtual knockout (KO) framework that leverages scRNA-seq data to systematically infer the functional consequences of gene perturbation. Its workflow proceeds in three steps: first, a denoised gene regulatory network (GRN) is constructed via scTenifoldNet; second, a virtual KO is implemented by setting all edges incident to the target gene to zero; and third, manifold alignment is used to compare the wild-type and perturbed networks, yielding a set of differentially regulated (DR) genes. We applied this approach to key candidate genes to anticipate their functional roles and map their downstream regulatory impact.

### 4.14. Genomic Risk Locus Annotation

Genomic risk loci for LC and HF were identified and annotated using the FUMA platform (https://fuma.ctglab.nl/snp2gene, accessed on 11 April 2026). GWAS summary files were uploaded, and FUMA performed preliminary QC by removing variants with missing data or poor quality metrics. A genome-wide significance threshold of *p* < 5 × 10^−8^ was used to define lead SNPs, which were then used to delineate independent risk loci.

### 4.15. Conditional Analysis of Genomic Risk Loci

To interrogate whether colocalized GWAS loci harbored independent secondary signals, conditional association analysis was performed using the COJO module within the GCTA software (v1.94.1) suite [40], conditioning on the lead variant at each locus. Variants with MAF below 0.0001 were excluded prior to analysis to ensure inclusion of low-frequency lead variants. Allele frequency data required for COJO were sourced from the 1000 Genomes Project.

### 4.16. Exon-Level Expression and Splicing Analysis of Prioritized Genes

Exon-level expression profiles of prioritized genes were retrieved from the GTEx portal (https://gtexportal.org/) to characterize transcript isoform diversity, structural features of individual transcripts, and tissue-specific expression patterns across human tissues.

## 5. Conclusions

By erecting a multi-layered integrative framework spanning genome-wide associ-ation data and single-cell transcriptomics, this study has systematically illuminated the shared genetic and molecular underpinnings of LC–HF comorbidity. Converging multi-dimensional evidence consistently implicates endothelial cells as the principal effector cell population and CRIM1 as the key candidate gene, each playing important roles in the shared pathological trajectory of LC and HF. This work is the first to computationally prioritize the endothelial cell–CRIM1 axis as a candidate component of the molecular machinery connecting LC and HF, providing fresh genetic evidence for cardiac–hepatic crosstalk and establishing a conceptual foundation for developing CRIM1-directed therapeutic strategies targeting this comorbidity.

## Figures and Tables

**Table 1:** Cell type enrichment of e/sQTL-mapped genes for LC and HF GWAS locus sets using ECLIPSER.

Trait	Tissue	Cell Type	Fold-Enrichment	Fold-Enrichment (95% CI)	*p*
LC	Liver	Hepatocytes	1.067	0.427, 1.839	0.431
HF	Heart	Endothelial cells	0.892	0.033, 4.359	0.542
HF	Heart	Monocyte	1.804	0.272, 5.339	0.231
HF	Heart	NK cell	2.176	0.329, 6.449	0.173
HF	Heart	Neutrophils	0.955	0.782, 0.999	0.995
HF	Heart	Smooth muscle cells	0.782	0.029, 3.812	0.590
HF	Heart	Tissue stem cells	1.401	0.213, 4.121	0.330

LC, Liver cirrhosis; HF, Heart Failure; CI, Confidence Interval.

## Data Availability

The datasets analyzed during the current study are available in the finngen database repository, https://www.finngen.fi/en (IDs: CHIRHEP_NAS and I9_HEARTFAIL, accessed on 12 April 2026). The datasets analyzed in the current study are available in the IEU OpenGWAS repository, https://opengwas.io/ (IDs: ebi-a-GCST90018826 and ebi-a-GCST009541, accessed on 17 April 2026). The datasets analyzed in the current study are available in the GEO repository, https://www.ncbi.nlm.nih.gov/geo/ (IDs: GSE312804 and GSE247468, accessed on 28 April 2026). The datasets analyzed in the current study are available in the GTEx repository, https://gtexportal.org/home/datasets (eQTL and sQTL summary statistics, accessed on 15 January 2026). The datasets analyzed in the current study are available in the MSigDB repository, http://www.gsea-msigdb.org/gsea/msigdb/collections.jsp, accessed on 10 December 2025 (Gene Ontology, Reactome, and KEGG gene sets). The datasets analyzed in the current study are available in the Mouse Genome Informatics (MGI) repository, http://www.informatics.jax.org/, accessed on 10 December 2025 (Mouse Phenotype Ontology gene sets).

## Supplementary Materials

The following supporting information can be downloaded at: https://www.mdpi.com/article/10.3390/ijms27177936/s1.

## Abbreviations

The following abbreviations are used in this manuscript:

LC	Liver Cirrhosis
HF	Heart Failure
GWAS	Genome-Wide Association Study
SNP	Single Nucleotide Polymorphism
MAF	Minor Allele Frequency
eQTL	Expression Quantitative Trait Locus
sQTL	Splicing Quantitative Trait Locus
gsMap	Genetically Informed Spatial Mapping of Cells for Complex Traits
scRNA-seq	Single-Cell RNA Sequencing
seismicGWAS	Seismic Genome-Wide Association Study Framework
ECLIPSER	—(see original method citation)
hdWGCNA	High-Dimensional Weighted Gene Co-Expression Network Analysis
WGCNA	Weighted Gene Co-Expression Network Analysis
eCAVIAR	Expression Causal Variants Identification in Associated Regions
fastenloc	Fast Enrichment Estimation Aided Colocalization Analysis
scTenifoldKnk	Single-Cell Tensor Decomposition-Based Virtual Knockout
FUMA	Functional Mapping and Annotation of Genome-Wide Association Studies
GCTA	Genome-Wide Complex Trait Analysis
COJO	Conditional and Joint Association Analysis
CRIM1	Cysteine-Rich Transmembrane BMP Regulator 1
BMP	Bone Morphogenetic Protein
LCP1	Lymphocyte Cytosol Protein 1
PTPRC	Protein Tyrosine Phosphatase Receptor Type C
CD45	Cluster of Differentiation 45 (encoded by PTPRC)
QC	Quality Control
UMI	Unique Molecular Identifier
PCA	Principal Component Analysis
UMAP	Uniform Manifold Approximation and Projection
TOM	Topological Overlap Matrix
GRN	Gene Regulatory Network
DR genes	Differentially Regulated Genes
KO	Knockout
GTEx	Genotype-Tissue Expression Project
GEO	Gene Expression Omnibus
GO	Gene Ontology
KEGG	Kyoto Encyclopedia of Genes and Genomes
MSigDB	Molecular Signatures Database
MGI	Mouse Genome Informatics
HALLMARK	Hallmark Gene Sets (MSigDB)
GO BP	Gene Ontology Biological Process
GO CC	Gene Ontology Cellular Component
RAAS	Renin–Angiotensin–Aldosterone System
NO	Nitric Oxide
LSECs	Liver Sinusoidal Endothelial Cells
CLPP	Colocalization Posterior Probability
RCP	Regional Colocalization Probability
LD	Linkage Disequilibrium
MAGMA	Multi-marker Analysis of GenoMic Annotation
SMAD	Small Mothers Against Decapentaplegic
sc-ST	Single-Cell Spatial Transcriptomics
